# Halophyte Litter Decomposition Shapes Soil Microbial Community Compositional Constancy by Regulating Resource Stoichiometry and Enzymatic Activity in a Microcosm Study

**DOI:** 10.1002/ece3.73871

**Published:** 2026-06-18

**Authors:** Yaqing Pan, Bahetijiang Ayala, Qing Wang, Zixuan Chen, Peng Kang

**Affiliations:** ^1^ Xinjiang Laboratory of Lake Environment and Resources in Arid Zone, College of Geographic Science and Tourism Xinjiang Normal University Urumqi China

**Keywords:** halophyte leaf quality, leaf decomposition, microbial community compositional constancy, microbial nutrient limitation, soil nutrients

## Abstract

A key knowledge gap exists in understanding how the decomposition of litter from different halophyte species influences microbial community dynamics in soils. This study addressed this gap through a 180‐day laboratory microcosm experiment investigating the effects of leaf litter decomposition from three halophytes (*Kalidium cuspidatum*, *Nitraria tangutorum*, and *Reaumuria songarica*) on soil biogeochemical properties, microbial dynamics, and community compositional constancy. The main research results indicate that at 180 days, the leaf mass loss (Mm) of the three halophytes reached 40.09%–42.89%, and the decomposition constants (*k*) were all < 0.2. Leaf total nitrogen, lignin, and carbon/nitrogen ratio directly regulated the decomposition process. Decomposition significantly increased soil nutrient pools, including total organic carbon (57.64%–100.12%), total nitrogen (51.92%–129.80%), dissolved organic carbon (44.35%–224.40%), and dissolved organic nitrogen (24.15%–238.58%), relative to bulk soil. Microbial carbon limitation increased by 21.81%–37.99%, while nitrogen limitation was alleviated, as evidenced by a 67.86%–92.28% increase in the vector angle of enzyme stoichiometry. These changes were driven by soil chemistry (explaining 45.47% of the variance) and microbial traits (42.31%–65.77%). Plant litter decomposition reshaped the structure of bacterial and fungal communities while reshaped the structure, which was linked to microbial biomass carbon, β‐glucosidase, and alkaline phosphatase (*p* < 0.05). Furthermore, partial least squares path modeling revealed that plant litter decomposition increased soil organic resources, thereby exacerbating microbial carbon limitation; yet, along with microbial biomass, it also influenced microbial community composition. These results underscore species‐specific litter effects on soil–microbe feedbacks in a controlled microcosm, emphasizing the role of resource stoichiometry and enzymatic activity in shaping microbial community in saline ecosystems.

## Graphical abstract

The results in the manuscript underscore the species‐specific litter effects on soil‐microbe feedbacks, emphasizing the role of resource stoichiometry and enzymatic activity in shaping microbial stability in saline ecosystems.

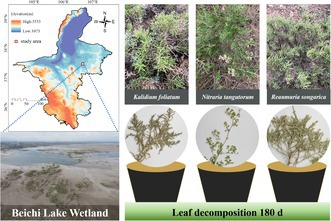

## 1 Introduction

Salt marsh ecosystems are particularly vulnerable and sensitive to water scarcity and high‐salinity stress in arid and semi‐arid regions (Carol et al. 2016; Xue et al. 2018; Pan et al. 2021). Halophytes are primary producers in these regions. Through their unique physiological and ecological adaptation strategies, halophytes form the structural foundation and functional core of salt marsh ecosystems (Zhao et al. 2011; Rozema and Schat 2013; Lu et al. 2023). The decomposition of plant litter serves as a crucial bridge linking plant production to soil processes, acting as the core biological process that drives nutrient cycling, including carbon and nitrogen, and energy flow within wetland soils (Jackson et al. 2017; Yu et al. 2025). Decomposed litter directly adds organic matter and nutrients to the soil. Their dissolved form, released during decomposition, along with the microenvironment, profoundly influences the composition, activity, and function of soil microbial communities (Riggs et al. 2015; Man et al. 2025; Wan et al. 2025). However, how the different species of halophytes and chemical properties of leaf litter regulate the effectiveness of soil resources, microbial metabolic limitations, and community compositional constancy remains unclear. Clarifying this process is crucial for understanding the material cycling mechanisms in salt marsh ecosystems in arid regions, predicting their responses to environmental changes, and implementing scientific ecological management.

Litter is the primary source of energy and nutrients for microorganisms, and its chemical properties, carbon‐to‐nitrogen (C/N) ratio and lignin content, are considered key factors in controlling decomposition rates and pathways (Augusto et al. 2015; Yang et al. 2025; Zhao et al. 2025). Generally, litter with low C/N ratios and lignin content decomposes rapidly, releasing available nutrients faster, whereas litter with high lignin content decomposes slowly, potentially leading to the accumulation of soil organic matter (Wan et al. 2025). In salt marsh wetlands, salt stress may inhibit microbial activity through osmotic stress and ion toxicity, potentially slowing the rate of litter decomposition (Zhai et al. 2020; Ni et al. 2024). However, the litter of salt‐tolerant halophytes has a unique chemical composition comprising compatible solutes and specific secondary metabolites, thereby altering its decomposition patterns in saline environments and changing its “resource effects” on soil microorganisms (Yadav et al. 2012; Ravi et al. 2020). Here, through a 180‐day controlled laboratory microcosm experiment, we addressed a significant knowledge gap regarding litter decomposition by multiple halophytes in arid‐salt marshes, which has been largely overlooked by previous studies that focused on single litterfall decomposition in non‐saline habitats (Casa et al. 2025; Liu et al. 2025). This gap concerns how such decomposition influences microbial nutrient acquisition strategies and community compositional constancy by altering soil resource pools and microbial biomass.

The capacity of a microbial community to resist environmental disturbances and maintain its structure and function is fundamental to ecosystem functioning (Yan et al. 2025; Zhi et al. 2025). The resource availability theory posits that ample resources help maintain high microbial diversity and functional redundancy, thereby supporting robust community structure and function (Delgado‐Baquerizo et al. 2016; Yuan et al. 2022). Conversely, resource limitations, such as carbon and nitrogen limitations, intensify competition among microorganisms, potentially leading to fluctuations in community composition (Zheng et al. 2022; Kang et al. 2025). As a key event, litterfall should theoretically alleviate resource limitations and enhance community functioning (Wang, Chen, et al. 2019; Wang, Liu, et al. 2019; Murúa and Gaxiola 2023). However, in habitats experiencing concurrent salt and drought stress, this process may become more complex. Although litter increases carbon sources and nutrients, potentially stimulating microbial growth and alleviating nutrient limitations (Men et al. 2025), decomposition processes may alter soil physicochemical properties (e.g., salinity and pH), and variations in litter chemical properties could lead to imbalanced resources (e.g., high C/N ratio exacerbates nitrogen limitation) (Wang et al. 2025; Wan et al. 2025). This imbalance exerts a selective pressure on microbial communities, affecting their composition and function (Bani et al. 2018; Wang and Kuzyakov 2024). Furthermore, bacteria and fungi, as distinct functional taxa involved in decomposition, respond differently to changes in resources.

Therefore, a significant knowledge gap remains concerning how the species‐specific chemical properties of halophyte litter directly control soil resource availability, microbial metabolic limitations, and the resultant dynamics of microbial communities. To address this gap, we conducted a 180‐day decomposition experiment with three halophytes (*K. cuspidatum*, *N. tangutorum*, and *R. songarica*) and tested the following hypotheses: (1) Initial litter chemistry determines decomposition rate; (2) Decomposition alters soil resource pools, thereby affecting microbial nutrient limitations; and (3) These changes in resources and limitations are the fundamental drivers of soil bacterial and fungal community structure and composition.

## 2 Materials and Methods

### 2.1 Plant Sample Collection and Experimental Design

Plant samples were collected from a single, representative location within the Beichi salt marsh wetland in Ningxia, China, to minimize site‐specific heterogeneity in substrate chemistry. The dominant plants were halophytes (e.g., *K. cuspidatum*, *N. tangutorum*, and *R. songarica*). Leaves were collected from at least 15 individual plants of each species, spaced at least 10 m apart, and pooled to create a composite sample for each species. In July 2024, leaf samples from these plants were collected separately, placed in sterile sealed bags, and transported to the laboratory. Subsequently, the leaves were washed with deionized water, heated in an oven at 65°C for fixation, and dried at 105°C until a constant weight was achieved.

**FIGURE 1:**
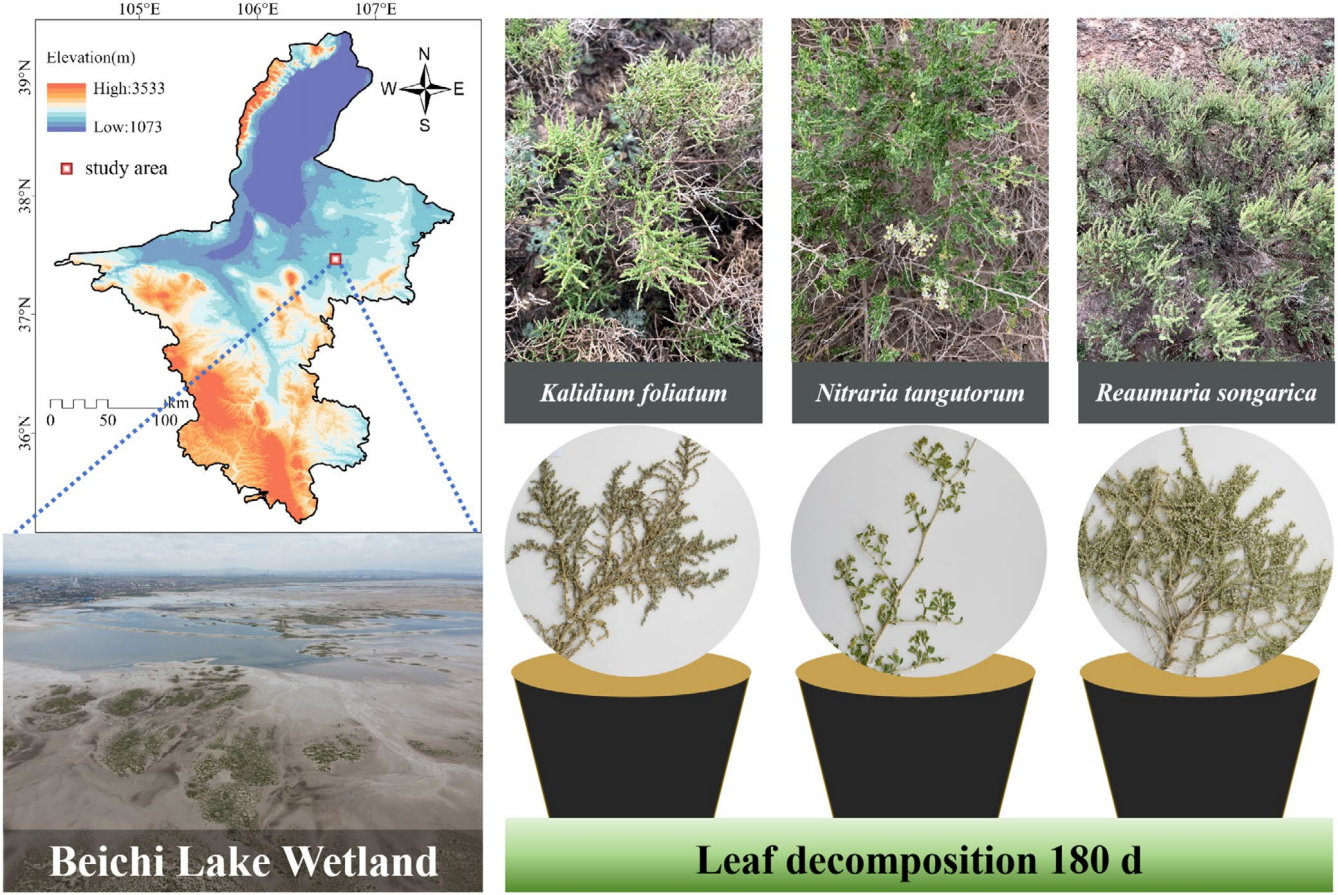
Sampling sites in Beichi salt marsh wetland in Ningxia.

Two grams of dried leaves from each halophyte were placed in separate nylon bags (10 cm × 10 cm, mesh size < 0.1 mm). After marking the bags, they were placed in plastic flowerpots (diameter, 15 cm) containing 1.5 kg of sandy soil. Sandy soil, collected from the same Beichi salt marsh wetland, was sieved (2 mm) and used as the substrate. Its properties were: total organic carbon content of 4.22 g·kg^−1^, total nitrogen content of 0.09 g·kg^−1^, and total phosphate content of 0.11 g·kg^−1^. Flowerpots containing *K. cuspidatum*, *N. tangutorum*, and *R. songarica* litter were labeled as KC, NT, and RS, respectively. Flowerpots containing only sand were used as controls and labeled BS. The experiment was conducted in a controlled laboratory incubator at a constant temperature of 25°C ± 1°C and a 12 h:12 h light/dark cycle. During the experiment, deionized water was sprayed to maintain the soil moisture at 40% of the maximum field capacity. The experiment was conducted for 180 days, and samples were collected and weighed on Days 30, 60, 90, and 180 to measure various indicators (Figure 1; Figure S1) (Drawing review No. GS (2024)0650).

Each treatment (BS, KC, NT, RS) consisted of 72 individual flowerpots in total (18 pots per treatment × 4 time points). At each sampling time (30, 60, 90, and 180 days), 18 flowerpots per treatment were destructively sampled. For each treatment and time point, the 18 pots were randomly assigned to 6 groups of 3 pots each. The soil from the 3 pots within a group was thoroughly mixed to create one composite sample (*n* = 6 per treatment per time point). The litter bags from each corresponding group were removed, washed, and dried to a constant weight to calculate the litter mass loss rate (Mm) and decomposition constant (*k*) (Olson 1963).

(1) $$\mathrm{Mm}\left(\%\right)=\left[\frac{\left(W_{o}-W_{t}\right)}{W_{o}}\right]\times 100\%$$

(2) $$k=-\frac{1}{t}\times \ln\frac{W_{t}}{W_{o}}$$

In the formula, *W*_0_ = 2.0 g, and *W*_t_ represents the weights measured at 30, 60, 90, and 180 days.

### 2.2 Statistical Analysis of Physical and Chemical Properties

Before conducting the experiment, the plant leaf samples were first washed with deionized water and then dried in a 65°C oven until a constant weight was achieved. Approximately 50 g of dried leaves were used for each chemical analysis, with mass determined using an analytical balance (precision 0.0001 g). Following grinding, the samples were used for subsequent measurements. The carbon (Leaf TC) and nitrogen (Leaf TN) contents of the leaves were determined using an elemental analyzer (Yang et al. 2018). The lignin content of the leaves was determined using the acetyl bromide‐ultraviolet spectrophotometry method. The sample powder was extracted with a neutral detergent to obtain cell wall residues. Subsequently, a 25% acetyl bromide‐acetic acid solution was added to the sample at 70°C in a water bath. After the reaction was terminated using a sodium hydroxide‐hydroxylamine solution, detection was performed (Freda et al. 2012; Kang et al. 2023).

At the experimental durations of 30, 60, 90, and 180 days, for each time period, 18 plants were selected from each treatment, and 3 plants were combined to form one representative sample. After removing the litter bags, soil from each composite pot group was collected aseptically, yielding six soil samples per treatment per time point. For high‐throughput sequencing, DNA was extracted from the 24 samples collected at the final 180‐day time point (4 treatments × 6 replicates). After the fresh soil samples were brought back to the laboratory, 20 g of air‐dried soil was mixed with 50 mL of deionized water following standard methods. Soil pH and EC were measured using a digital pH meter (LeCid PHS‐3E, Shanghai, China) and a conductivity meter (LeCid DDS‐307, Shanghai, China), respectively (Bao 2000).

The 24 soil samples (from Day 180) were then sieved and air‐dried. Total carbon (TC) and total organic carbon (TOC) were analyzed using a Shimadzu TOC‐V Series SSM‐5000A analyzer (Shimadzu, Japan). Soil total nitrogen (TN) was determined by dry combustion using an elemental analyzer (Vario MACRO cube, Elementar, Germany) (Wang, Chen, et al. 2019; Wang, Liu, et al. 2019). Total phosphorus (TP) was measured via sulfuric acid digestion, followed by molybdenum blue colorimetry (Reed and Martens 1996). Dissolved organic carbon (DOC) and total dissolved nitrogen (TDN) were extracted with 0.5 M K_2_SO_4_ solution and quantified using a TOC analyzer (Kalbitz et al. 2000). Total inorganic nitrogen (TIN), including NH^+^_4_‐N, NO^−^‐N, and NO_2_^−^‐N, was measured. Dissolved organic nitrogen (DON) was calculated as DON = TDN—TIN (Jones and Willett 2006).

### 2.3 Soil Extracellular Enzyme Activity

The soil extracellular enzyme activities involved in major nutrient cycles were determined, including β‐1,4‐glucosidase (BG) for carbon acquisition, two enzymes for nitrogen acquisition, β‐1,4‐N‐acetyl‐glucosamidase (NAG) and leucine aminopeptidase (LAP), and alkaline phosphatase (AKP) for phosphorus acquisition. Enzyme activities were measured using 4‐methylumbelliferone‐linked substrates for BG, NAG, and AKP, and L‐Leucine‐7‐amido‐4‐methylcoumarin for LAP. Fresh soil samples were homogenized in buffer solutions, with BG and NAG assayed in acetate buffer and LAP and AKP assayed in Tris buffer, respectively. Aliquots of soil suspension (150 μL) and substrate solution (50 μL) were dispensed into black 96‐well microplates and incubated in the dark. Reactions were terminated by adding 10 μL of NaOH, and fluorescence was measured using a microplate reader, following established protocols (Marx et al. 2001; German et al. 2011).

### 2.4 Soil Microbial Biomass

Soil microbial biomass was extracted using the chloroform fumigation method. Soil TOC and TN were determined by extracting fumigated and unfumigated soil with K_2_SO_4_ (Vance et al. 1987; Brookes et al. 1985)

(3) $$\mathrm{MBC}=\frac{\left[{\mathrm{DOC}}_{\left(\text{fumigated}\right)}-{\mathrm{DOC}}_{\left(\text{unfumigated}\right)}\right]}{0.45}$$

(4) $$\mathrm{MBN}=\frac{\left[{\mathrm{TN}}_{\left(\text{fumigated}\right)}-{\mathrm{TN}}_{\left(\text{unfumigated}\right)}\right]}{0.54}$$

where 0.45 and 0.54 are the extraction efficiency coefficients for fumigation extraction of MBC and MBN.

### 2.5 Data Processing for Microbial Nutrient Limitation

This analysis determines the type and intensity of microbial nutrient limitation (Sinsabaugh et al. 2009; Moorhead et al. 2016). Vector Length (L): Reflects the degree of carbon limitation (C limitation). A greater length indicates a stronger C limitation.

(5) $$L=\sqrt{{\left(\frac{\mathrm{BG}}{\mathrm{BG}+\mathrm{NAG}+\mathrm{LAP}}\right)}^{2}+{\left(\frac{\mathrm{BG}}{\mathrm{BG}+\mathrm{AKP}}\right)}^{2}}$$

Vector Angle (θ): Indicates relative N/P limitation. An angle approaching 0° signifies nitrogen limitation (N limitation), whereas an angle approaching 90° signifies phosphorus limitation (P limitation)

(6) $$\theta =\text{Degrees ATAN}2\;\left[\frac{\mathrm{BG}}{\mathrm{BG}+\mathrm{AKP}},\frac{\mathrm{BG}}{\mathrm{BG}+\mathrm{NAG}+\mathrm{LAP}}\right]$$

### 2.6 Soil DNA Extraction and High‐Throughput Sequencing

Total soil DNA was extracted from fresh soil samples (*n* = 24 from Day 180) using a cetyltrimethylammonium bromide‐based method. Soil samples were homogenized under liquid nitrogen, and impurities were removed using isopropanol precipitation, followed by DNA purification. The V3–V4 regions of bacterial 16S rRNA gene were amplified from all qualified samples using the primer pair 341F (5′‐CCTAYGGGRBGCASCAG‐3′) and 806R (5′‐GGACTACNNGGGTATCATAT‐3′) (Caporaso et al. 2011). Simultaneously, primers ITS1 (5′‐CTTGGTCATTTAGAGGAAGTAA‐3′) and ITS2 (5′‐GCTGCGTTCTTCATCGATGC‐3′) were used to amplify the ITS1‐1 gene region (Schoch et al. 2012). The amplicons were sequenced using an Illumina NovaSeq 300 platform. Raw sequence reads were merged using FLASH (version 1.2.11) (Magoč and Salzberg 2011). Chimeric sequences were removed using USEACH (Haas et al. 2011), and high‐quality sequences were denoised using the DADA2 plugin in QIIME (Version 2.0) to generate amplicon sequence variants (ASVs) (Callahan et al. 2016). In this study, the representative sequences of each ASV were annotated to obtain the diversity (Shannon) and richness (ACE) indices of the bacterial and fungal communities (Bolyen et al. 2019; Bokulich et al. 2018).

### 2.7 Data Analyzes

All statistical analyzes were performed using R version 4.1 (Racine 2012). A three‐way analysis of variance (ANOVA) with Treatment, Time, and their interaction as fixed factors was initially used to assess overall effects on decomposition rates and soil properties. For endpoint comparisons (Day 180), a one‐way ANOVA followed by Duncan's post hoc test was used to compare treatment means. Data were visualized using the “ggplot2” package. Redundancy analysis (RDA) was performed on the initial mass and decomposition rate of the three halophyte plant leaves (Dray et al. 2012). For multivariate analyzes, Permutational Multivariate Analysis of Variance (PERMANOVA) with 999 permutations was used to test for significant differences in community structure (Bray‐Curtis distance) among treatments. Changes in soil bacterial and fungal communities were analyzed using Non‐Metric Multidimensional Scaling (NMDS) with the ‘vegan’ package. The relationships between microbial diversity parameters and soil properties, microbial traits, and metabolic limitations were displayed using heatmaps generated with the “pheatmap” package (Sunagawa et al. 2015).

The composition and transitions of the bacterial and fungal communities were visualized using the “ggalluvial” package. Microbial community compositional constancy (MCCC) was assessed as the resistance to change, quantified as 1‐AVD (Average Variability Degree), where AVD is the average Euclidean distance of community structure (based on ASV relative abundance) (Xun et al. 2021). Random forest analysis was used to assess the importance of soil physical, chemical, and microbial properties on microbial metabolism (Jiao et al. 2018). Variable importance was estimated by the permutation method (%IncMSE), and the model's predictive performance was assessed using 10‐fold cross‐validated *R* ^2^. Finally, to evaluate the effects of leaf decomposition on soil resources, microbial biomass, and microbial metabolic limitations on community composition and compositional constancy, we developed a partial least squares path model (PLS‐PM) using the plspm package in R software (Sanchez et al. 2022). In the PLS‐PM model, we used the reciprocals of Mm and *k* as the leaf decomposition indicators, DOC and DON as the indicators of soil resources, and MBC and MBN as the indicators of microbial biomass. C and N limitations serve as indicators of microbial nutrient limitation; among them, N limitation was obtained by calculating the reciprocal of the vector angle. The dominant phylum identified through random forest analysis represents the dominant phylum. MCCC was derived from the 1 minus AVD parameters of bacteria and fungi.

## 3 Results

### 3.1 Relationship Between Leaf Litter Mass and Decomposition in Three Halophytes

**FIGURE 2:**
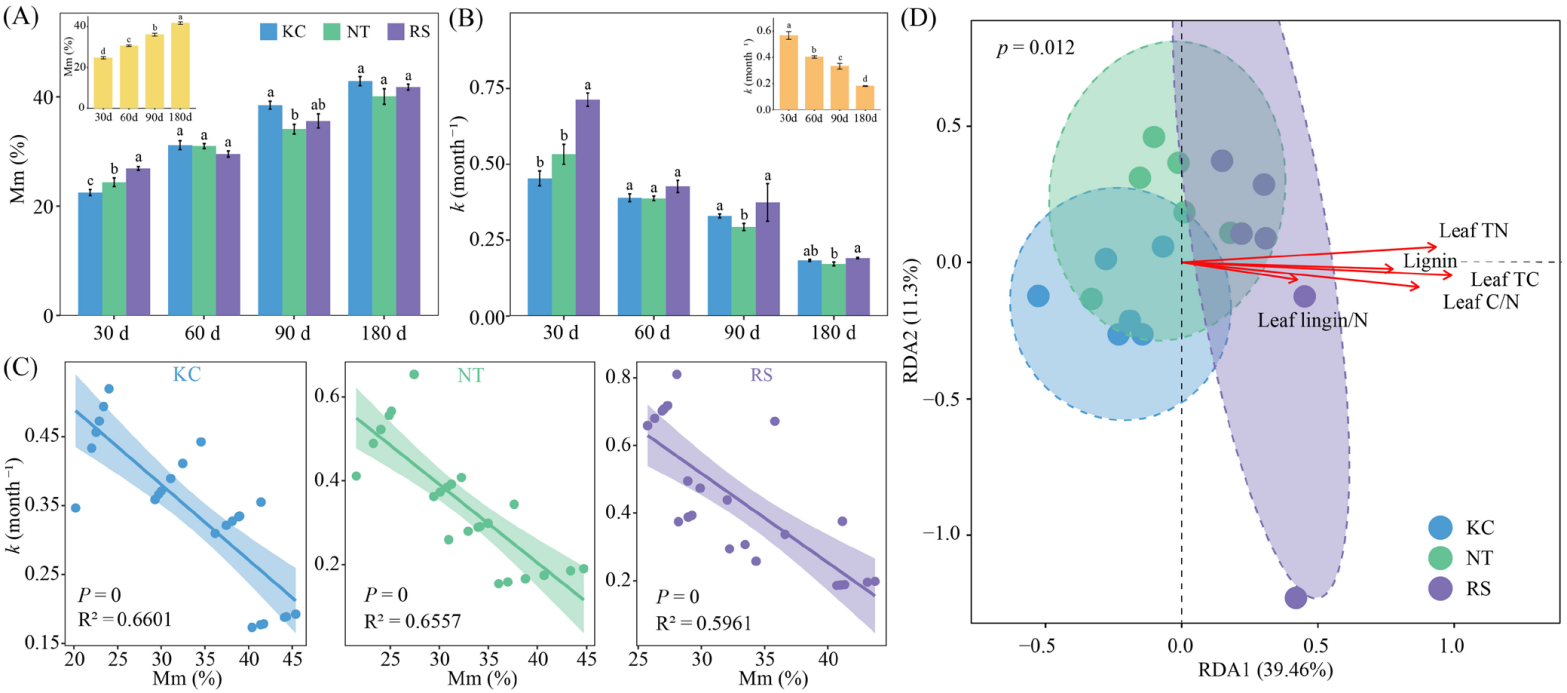
Leaf Mm and *k* values of three halophytes at 30, 60, 90, 180 days (A, B), and the linear relationship between Mm and *k* values (C), as well as the RDA analysis of leaf quality and Mm and *k* values (D).

The chemical properties of *K. cuspidatum*, *N. tangutorum*, and *R. songarica* leaves grown in salt marsh wetlands differed significantly. The TC, TN, and lignin contents of *R. songarica* leaves were higher than those of *K. cuspidatum* and *N. tangutorum*, whereas no significant differences in the leaf C/N and lignin‐to‐N ratios were observed (*p* < 0.05) (Figure S2). *R. songarica* leaves exhibited the greatest mass loss at Day 30, whereas *K. cuspidatum* leaves exhibited the highest cumulative mass loss at Day 90. Among the three halophytes, there leaves had the highest Mm (40.09%–42.89%) at Day 180, and the *k* values of all three halophytes at Day 180 were lower than 0.2 (*p* < 0.05) (Figure 2A,B). The leaf Mm and *k* values of the three halophytes exhibited a clear linear relationship (*p* = 0) (Figure 2C). Furthermore, leaf TN, lignin, leaf TN, and leaf C/N all had a direct correlation with the changes in Mm and *k* of the leaves (*p* = 0.012) (Figure 2D).

### 3.2 Effects of Leaf Decomposition on Soil Physicochemical Properties, Microbial Biomass, and Extracellular Enzyme Activity

After 180 days of leaf decomposition by halophytes, the EC of the soil increased, with the *K. cuspidatum* soil sample showing a 33.84% increase, whereas the *N. tangutorum* sample showed a 31.51% increase compared with the BS soil sample. The TOC, TN, DOC, and DON contents of the soil also increased significantly after leaf decomposition. Compared with those of the BS soil sample, the TOC of the *K. cuspidatum*, *N. tangutorum*, and *R. songarica* soil samples increased by 57.64%, 62.35%, and 100.12%, respectively; the TN increased by 51.92%, 71.44%, and 129.80%, respectively; the DOC increased by 44.35%, 70.88%, and 224.40%, respectively; and the DON increased by 24.15%, 62.36%, and 238.58%, respectively (*p* < 0.05) (Table S1).

In microbial biomass, leaf decomposition increased the MBC and MBN content to varying degrees. The soil MBC of *K. cuspidatum*, *N. tangutorum*, and *R. songarica* increased by 42.85%, 63.03%, and 89.92%, respectively, compared to the BS. The MBN increased by 43.53%, 52.94%, and 69.41%, respectively (*p* < 0.05) (Table S1). Additionally, extracellular enzyme activity significantly increased after leaf decomposition. Compared with those in the BS soil sample, the BG activities in the *K. cuspidatum*, *N. tangutorum*, and *R. songarica* soil samples increased by 202.68%, 220.08%, and 321.25%, respectively, while the AKP activities increased by 109.57%, 128.25%, and 138.14%, respectively (*p* < 0.05) (Table S1).

### 3.3 Effects of Leaf Decomposition on Soil Microbial Nutrient Limitation

**FIGURE 3:**
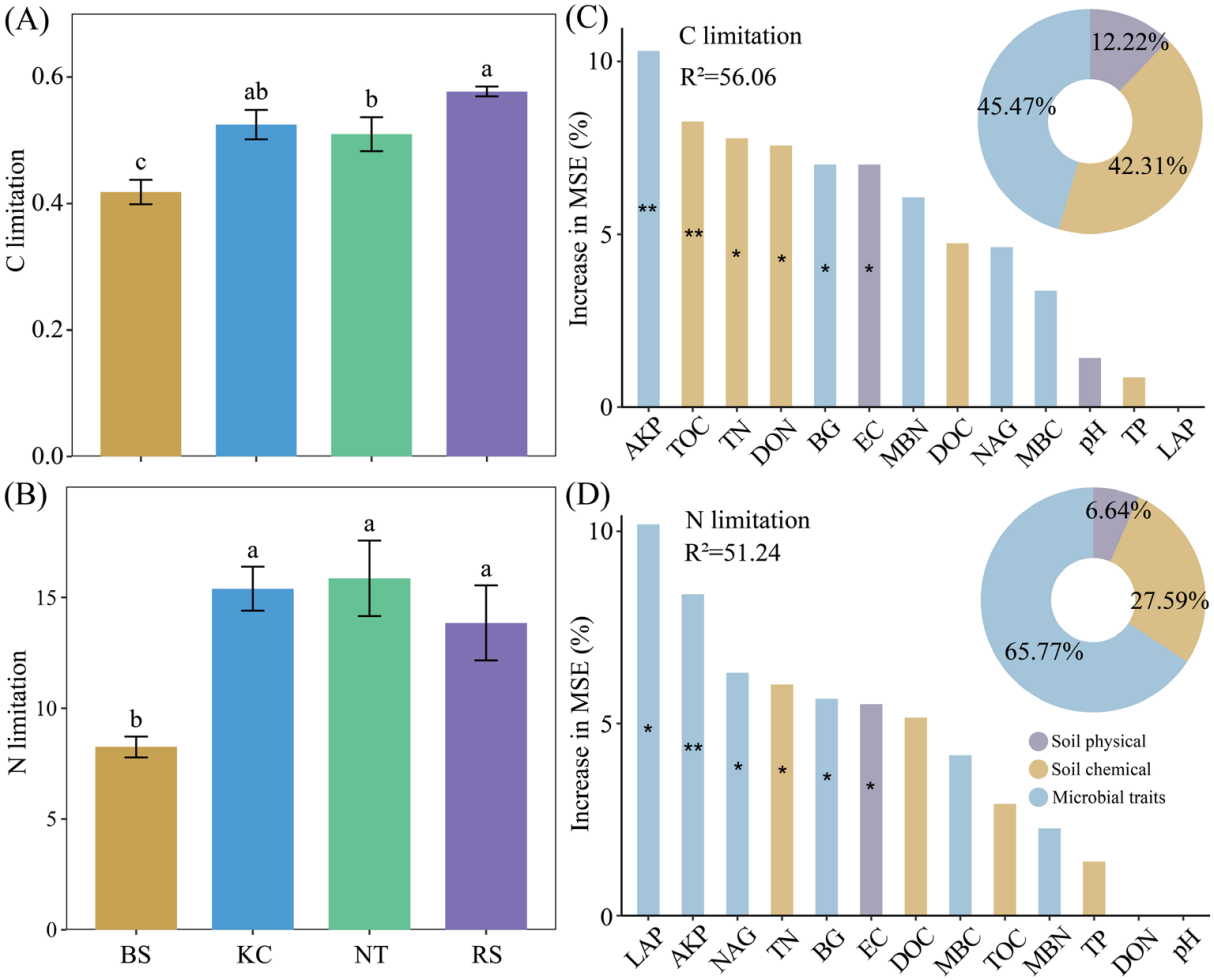
Microbial nutrient limitation (A, B) under leaf decomposition of different halophyte and the explanatory rates of soil physicochemical properties, microbial biomass, and extracellular enzyme activity (C, D).

After 180 d of leaf decomposition, microbial C limitation increased significantly (*p* < 0.05). Concurrently, the vector angle increased, indicating a significant shift from N limitation decreased in all litter‐treated soils compared to the control (*p* < 0.05) (Figure 3A,B). Compared with those in the BS soil sample, C limitation in the *K. cuspidatum*, *N. tangutorum*, and *R. songarica* soil samples increased by 25.51%, 21.81%, and 37.99%, respectively, whereas the vector angle increased by 86.60%, 92.28%, and 67.86%, respectively (*p* < 0.05) (Figure 3A,B). Random forest analysis revealed that AKP, TOC, TN, and DOC had high explanatory rates for C limitation, with soil chemical properties (45.47%) and microbial traits (42.31%) contributing significantly to the total explanatory rate. The LAP, AKP, NAG, TN, and BG had high explanatory rates for the shift in N limitation, with microbial traits accounting for 65.77% of the total explanatory rate (Figure 3C,D).

### 3.4 Effects of Leaf Decomposition on Soil Microbial Community Diversity and Structure

**FIGURE 4:**
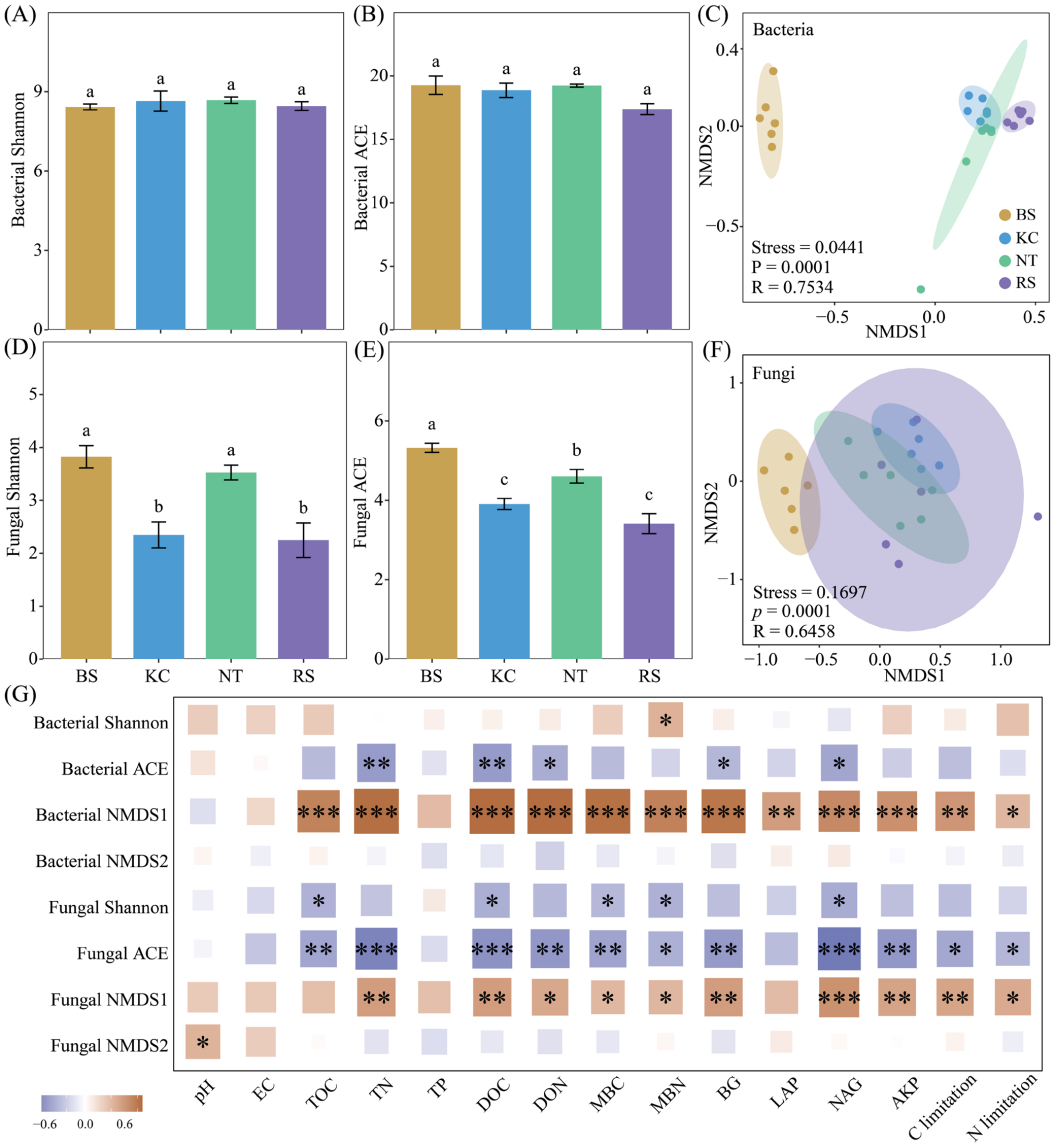
α and β diversity of soil bacterial (A–C) and fungal (D–F) communities under leaf decomposition of different halophyte, as well as their correlations with soil physicochemical properties, microbial biomass, extracellular enzyme activity, and microbial nutrient limitation (G). Different lowercase letters indicate significant differences. * *p* < 0.05, **p < 0.01, *** *p* < 0.001.

The decomposition of halophyte leaves had no significant effect on the diversity and richness of soil bacteria; however, it significantly altered the structure of the soil bacterial community. NMDS analysis revealed significant separation of bacterial (stress = 0.18, *R* ^2^ = 0.42, *p* = 0.001) and fungal (stress = 0.16, *R* ^2^ = 0.44, *p* = 0.001) community structures among the four treatments at Day 180 (Figure 4A–C). In contrast, the decomposition of *K. cuspidatum* and *R. songarica* leaves markedly reduced the diversity and richness of the soil fungal community and changed the structure of the fungal community (Figure 4D–F). Further analysis revealed that the presence of the bacterial and fungal communities in NMDS1 was positively correlated with TN, DOC, DON, MBC, MBN, BG, NAG, AKP, C, and N limitation, whereas the presence of the fungal community ACE was negatively correlated with these parameters (Figure 4G).

**FIGURE 5:**
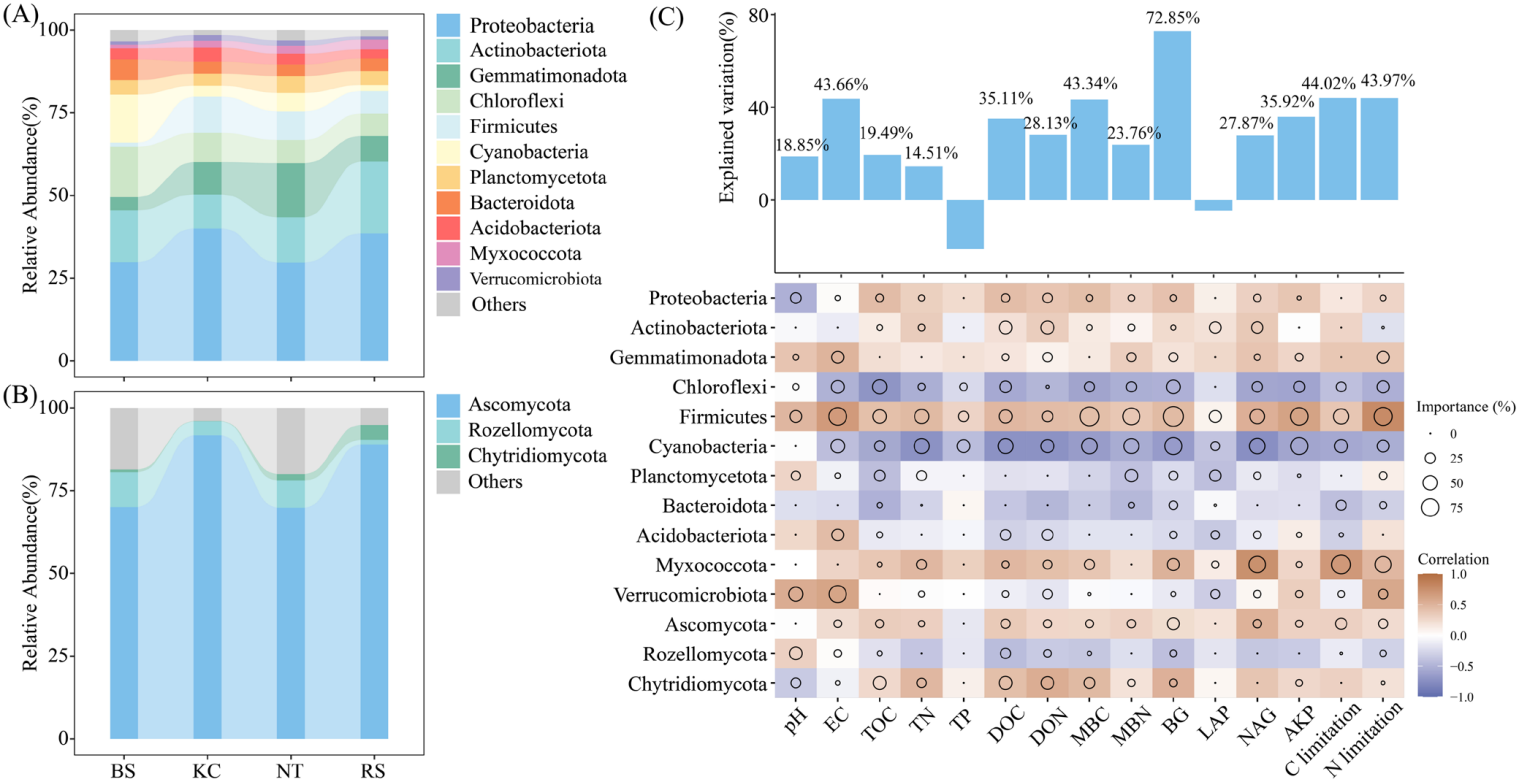
Soil bacterial (A) and fungal (B) community composition under leaf decomposition of different halophyte, and the explanatory rates of soil physicochemical properties, microbial biomass, extracellular enzyme activity, and microbial nutrient limitation on dominant phyla (C).

In this study, the soil bacterial community was primarily composed of Proteobacteria, Actinobacteriota, Gemmatimonadota, Chloroflexi, and Firmicutes. The fungal community was composed of Ascomycota, Rozellomycota, and Chytridiomycota (Figure 5A,B). Random forest analysis identified the primary factors influencing changes in the structure of soil bacterial and fungal communities. Among the parameters examined, BG (72.85%), C limitation (44.02%), N limitation (43.97%), EC (43.66%), MBC (43.34%), DOC (35.11%), and AKP (35.92%) exhibited relatively high explanatory rates (Figure 5C).

### 3.5 Effects of Leaf Decomposition on Soil Microbial Community Compositional Constancy

**FIGURE 6:**
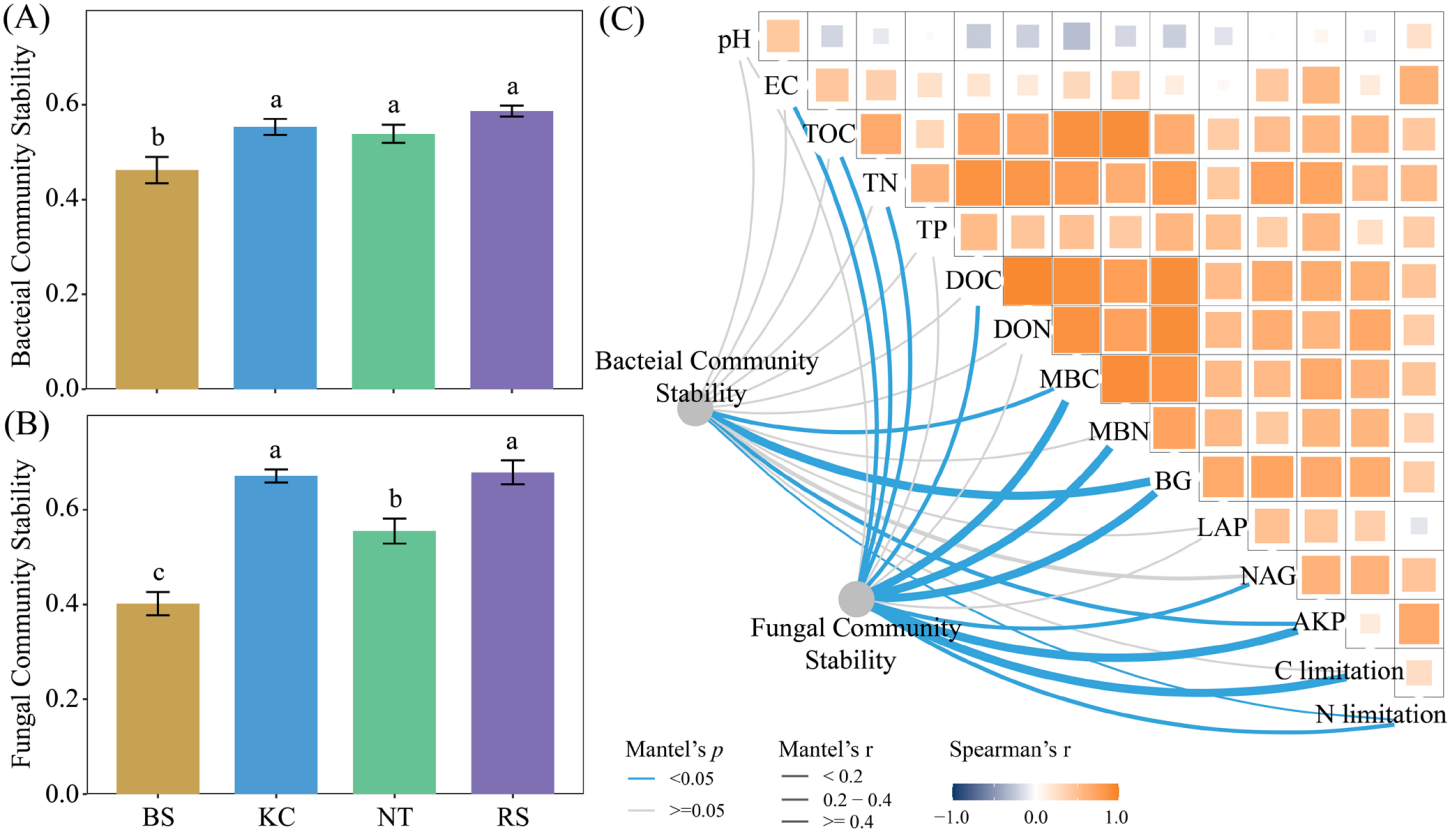
The stability of soil bacterial (A) and fungal (B) communities under leaf decomposition of different halophyte, and the Mantel analysis (C) of their relationship with soil physicochemical properties, microbial biomass, extracellular enzyme activity, and microbial nutrient limitation.

Decomposition of halophyte leaves altered the composition of soil bacterial and fungal communities to varying degrees (*p* < 0.05) (Figure 6A,B). Mantel analysis indicated that bacterial community structure was influenced by MBC, MBN, BG, NAG, AKP, and N limitation. Soil physicochemical properties, microbial biomass, extracellular enzyme activity, and nutrient limitation all affected fungal community structure (Figure 6C). C and N limitation were linearly correlated with bacterial community compositional constancy. Similarly, they were linearly correlated with fungal community compositional constancy (Figure S3). Furthermore, random forest analysis indicated that Planctomycetota, Firmicutes, and Proteobacteria exhibited the highest explanatory rates for the compositional constancy of the bacterial community (Figure S4A), whereas Ascomycota exhibited the highest explanatory rate for the compositional constancy of the fungal community (Figure S4B).

**FIGURE 7:**
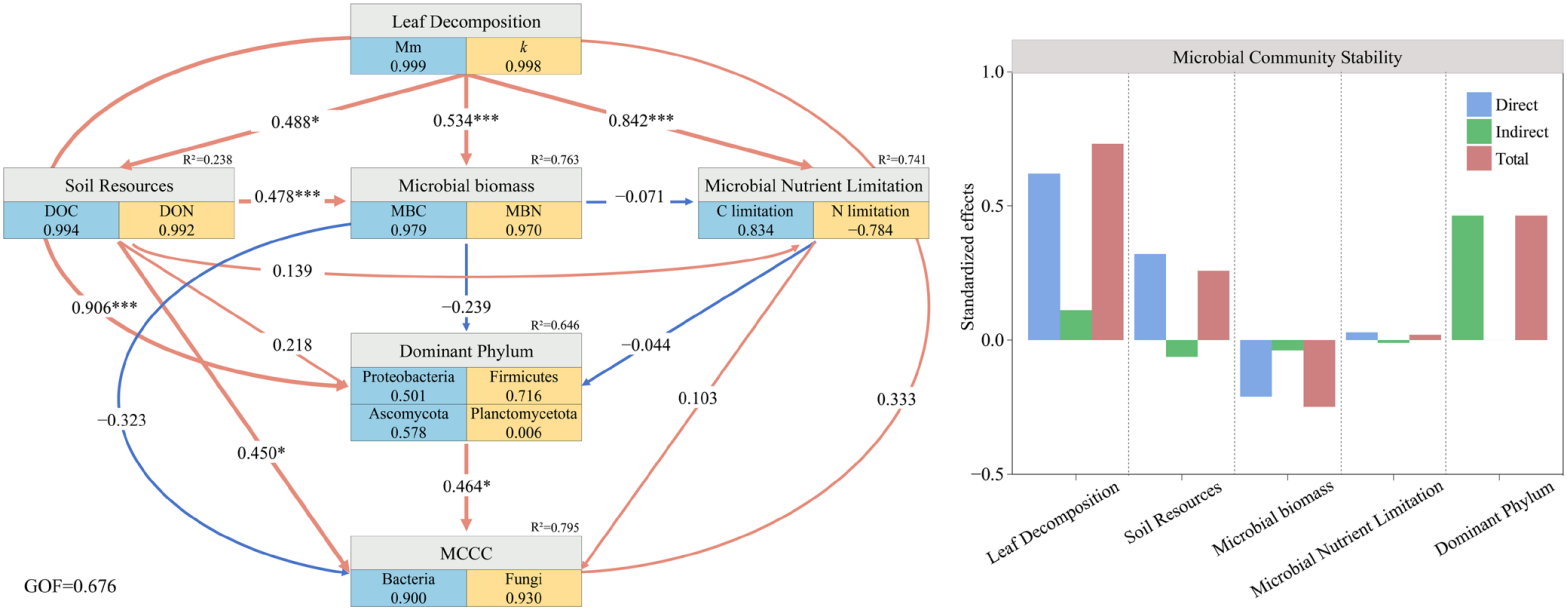
Soil resources, microbial biomass, microbial nutrient limitation, dominant phylum pathways for the partial least squares path model analysis of microbial community stability under leaf decomposition of different halophyte.

The PLS‐PM results revealed that leaf decomposition had a significant positive effect on soil resources (path coefficient = 0.488, *p* < 0.05), microbial biomass (path coefficient = 0.534, *p* < 0.001), and microbial nutrient limitation (path coefficient = 0.842, *p* < 0.001). Soil resources, in turn, had a strong positive effect on microbial biomass (path coefficient = 0.906, *p* < 0.001) and a direct positive effect on MCCC (path coefficient = 0.450, *p* < 0.05). In addition, changes in the main bacterial phyla also had a direct positive effect on MCCC (path coefficient = 0.464, *p* < 0.05) (Figure 7).

## 4 Discussion

### 4.1 Differences in Chemical Properties of Halophyte Leaves Drove Decomposition Dynamics and Affected Soil Resources

Halophytes are influenced by the regional water‐salt gradient, and their distribution and differences in their species are decisive factors in determining the chemical properties of their leaves (Sun et al. 2016; Zhai et al. 2020). In this study, *R. songarica* leaves exhibited the highest mass loss during the initial decomposition stage (Day 30), which might be related to their high TN content. In nutrient‐poor sandy soils, the high initial TN content of *R. songarica* leaves may have provided a sufficient nitrogen source for the early colonization of microbial communities, thus alleviating nitrogen limitation in the barren soil and promoting initial microbial activity and subsequent quality loss (Martiny and Vivanco 2025). As decomposition progressed to 90 days, the cumulative mass loss of *K. cuspidatum* surpassed that of the other species, potentially due to its lower initial C/N ratio supporting sustained microbial activity over the mid‐term. By 180 days, the remaining mass fractions of the three halophytes converged with similarly low decomposition rate constants (*k* < 0.2) (Sun et al. 2022; Yu et al. 2025). This dynamic process shows that litter decomposition under multiple abiotic stresses constitutes a multistage continuum. Litter with distinct chemical properties creates unique decomposition trajectories by promoting the growth of dominant microbial taxa and regulating the enzymatic systems that govern different decomposition stages (Mao et al. 2025).

After 180 days of decomposition, halophyte leaves had a significant impact on soil physicochemical properties. The decomposition of each halophyte markedly increased the soil EC, with *K. cuspidatum* and *N. tangutorum* increasing the EC the most (> 30%). This finding directly confirms that halophyte litter is a significant source of soil salinity (Cotrufo et al. 2015). Although microbial activity is usually inhibited in high‐salinity environments, in this study, all halophyte litter significantly increased TOC, TN, DOC, and DON, with *R. songarica* litter increasing these parameters the most. This finding indicates that the decomposition of the halophyte litter released carbon and nitrogen, which promoted microbial growth and outcompeted the inhibitory effect of salt mineralization, particularly in *R. songarica*, owing to its higher initial TC and TN contents and faster decomposition rate at the early stage (Gill et al. 2021; Martínez‐García et al. 2021).

### 4.2 Halophyte Litter Decomposition Regulated the Dynamics of Nutrient Limitation of Soil Microbes

The concurrent increase in MBC and MBN further corroborates the nutrient‐induced growth stimulation of halophyte litter on microbial communities (Lyu et al. 2023). Halophyte litter serves as a substrate, providing not only a direct carbon source but also releasing DOC and DON, which are more readily assimilated by microorganisms (Qiang et al. 2024). The activities of extracellular enzymes were significantly increased. In the *R. songarica* soil sample, BG and AKP activities increased by 321.25% and 138.14%, respectively, compared to those in the control soil sample. This finding indicates that litter decomposition strongly stimulated the capacity of microbial mineralization of carbon (Chen, Bai, et al. 2025; Chen, Cao, et al. 2025; Xie et al. 2025). The increase in enzyme activities might be driven by two factors: the dissolved organic matter released from the litter that directly induced enzyme synthesis and the expansion of microbial biomass that caused the “scale effect” of enzyme production (Błońska et al. 2021; Liu et al. 2021).

After 180 days of leaf decomposition, microbial carbon limitation increased significantly, with the greatest increase observed for *R. songarica* (37.99%), suggesting that interspecific differences in litter chemical properties modulate microbial resource allocation strategies (Men et al. 2025). Random forest analysis further indicated that C limitation was primarily co‐regulated by the availability of soil carbon and nitrogen in a matrix (e.g., TOC, TN, and DOC) and microbial functional traits (e.g., AKP), with both factors explaining nearly 90% of the variance. This finding highlights the close relationship between soil physicochemical properties and microbial metabolic activity (Abay et al. 2024; Kang et al. 2025). The high explanatory rate of AKP for carbon limitation may reflect the synergy of carbon–phosphorus metabolism in saline environments, where microorganisms must simultaneously consume carbon and phosphorus to sustain enzyme synthesis (Wang et al. 2024).

In terms of the N limitation, microbial traits (e.g., LAP, NAG, and BG) contributed to ≤ 65.77% of the variance, which significantly exceeded the contributions of soil chemical factors. This finding suggests that microorganisms in saline environments may alleviate N limitation by upregulating the activities of N and P hydrolytic enzymes. As N limitation decreases, the increased activity of LAP and BG reflects a shift in metabolic investment (Liu et al. 2024). The decomposition of halophyte litter may enhance the microbial community's nitrogen acquisition capacity by altering the stoichiometric ratio of available nutrients, thereby reshaping nutrient limitation patterns in the soil. This discovery provides new insights into the feedback mechanisms between the transformation of organic matter and microbial metabolism in saline ecosystems (Zhang, Luo, et al. 2025; Zhang, Zhai, et al. 2025).

### 4.3 Halophyte Litter Decomposition Reshaped Soil Microbial Communities

Although the decomposition of halophyte leaves did not significantly alter the diversity and richness of soil bacterial communities, it redistributed functional taxa and reshaped the bacterial community structure. This finding suggests that bacterial communities adapt to microenvironmental changes triggered by halophyte litter through interspecific substitution or functional redundancy, resulting in the compositional constancy of their overall diversity (Huang, Bao, et al. 2024; Huang, Li, et al. 2024). In contrast, the decomposition of *K. cuspidatum* and *R. songarica* leaves significantly reduced fungal community diversity and richness and reconfigured fungal community structure. This finding suggests that fungi are more sensitive than bacteria to halophyte litter, with certain taxonomic groups potentially undergoing selective elimination due to intensive competition for carbon use and salt–nutrient stress (Benito‐Carnero et al. 2021).

More importantly, the parameters of the bacterial and fungal communities (NMDS1) were positively correlated with the availability of carbon and nitrogen in the soil (DOC, DON, and TN), microbial biomass (MBC and MBN), and enzyme activities (BG, NAG, and AKP), whereas the fungal ACE index was negatively correlated with these parameters. This contradiction may be due to the different resource utilization strategies of bacteria and fungi in saline habitats. Enhanced nutrient availability (TN and DOC) and microbial metabolic intensity (AKP and BG) resulting from litter decomposition conferred competitive advantages to specific microbial groups (a transition from oligotrophs to copiotrophs), leading to a shift in the community structure toward highly active taxa (Zheng et al. 2021; Chen, Bai, et al. 2025; Chen, Cao, et al. 2025). However, resource enrichment caused a decline in fungal diversity owing to the “biological filtration effect”. High‐nutrient environments increase competition between fungal species, resulting in the dominance of fungal groups, such as Ascomycota, that thrive on substrates with a high carbon‐to‐nitrogen ratio, thereby compressing the ecological niches of other taxa (Wang, Chen, et al. 2019; Wang, Liu, et al. 2019; Yang et al. 2025).

Random forest analysis revealed that BG, which accounted for 91.16% of the explanatory rate, was the primary factor restructuring the microbial community during halophyte litter decomposition. This finding highlights the role of carbon hydrolases in regulating microbial community assembly. BG may restructure the bacterial community by directly participating in the cellulose degradation of halophyte litter (Xing et al. 2024). Enhanced BG activity indicates an increase in carbon resource availability, directing the accumulation of copiotrophic taxa in the microbial community (Zheng et al. 2021). The high explanatory rates of DOC (63.78%) and MBC (63.21%) further confirmed that the availability of carbon resources shaped the community structure. Increased DOC from halophyte litter altered soil carbon stoichiometry, thereby shifting the growth strategy of microbes from oligotrophic to copiotrophic (Wang et al. 2025), whereas MBC enhanced the intensity of microbial interactions, thereby indirectly influencing the composition of the microbial community (Elias et al. 2024).

The results of this study showed that AKP and DON formed a synergistic regulatory network with carbon (45.39%) and the N limitation (45.61%), thus contributing to 56.26% and 55.28% of the community structure, respectively. This finding indicates that nitrogen and carbon metabolisms are tightly coupled in saline environments, and that the coordinated variation of AKP and DON may reflect microbial strategies for acquiring both N and P under N‐limited conditions. It also suggests that the increased AKP activity was not solely an indicator of phosphorus demand but was also linked to carbon dynamics, as carbon serves as the substrate for enzyme synthesis and is consumed during this process. Consequently, taxa with efficient nutrient acquisition capabilities (i.e., Actinobacteriota) were selected (Singh et al. 2025).

### 4.4 Halophyte Litter Decomposition Enhanced Microbial Compositional Constancy

Our results suggest that, within the context of this 180‐day controlled experiment, halophyte litter decomposition was associated with an increase in the compositional constancy of both bacterial and fungal communities (Figure 6A,B). This indicates that the added resources may have promoted functional redundancy or keystone taxa that buffer the community against perturbation. However, the driving mechanisms differed significantly. The enhanced compositional constancy of the bacterial community may be explained by the redundancy of functional taxa and ecological strategies of key bacterial phyla (Planctomycetota, Firmicutes, and Proteobacteria) (Huang, Bao, et al. 2024; Huang, Li, et al. 2024). For example, Proteobacteria, a typical copiotrophic phylum, may rapidly respond to carbon sources from litter by increasing its metabolic activity, thereby buffering environmental disturbances (Wang et al. 2025). The specificity of Planctomycetota in complex carbon metabolism, together with the stress tolerance of Firmicutes, may serve as resistance mechanisms of the bacterial community against disturbances (Zhang, Luo, et al. 2025; Zhang, Zhai, et al. 2025). In contrast, although the compositional constancy of the fungal community increased, the low explanatory rate (7.5%) for halophyte litter decomposition suggested that abiotic factors, such as unmeasured biological interactions or microhabitat heterogeneity, played a dominant role. Ascomycota, a dominant phylum with a high explanatory rate, may suppress community fluctuations by occupying a dominant ecological niche, owing to its strong competitiveness for resources and its high salt tolerance (Zheng et al. 2021; Luo et al. 2023; Shepherd et al. 2025). The linear relationship between microbial nutrient limitations and the compositional constancy of the microbial community further revealed the effect of nutrient limitation on the regulation of enzymatic pathways. Increased C limitation forced microorganisms to rely on extracellular enzymes (BG and AKP) to decompose complex organic matter, thereby promoting metabolic division and cooperation among functional taxa and enhancing the overall robustness of the community (Kang et al. 2025).

The PLS‐PM analysis indicated that halophyte litter decomposition regulates soil microbial compositional constancy through a cascading pathway involving resource addition, nutrient limitation, and community restructuring. It directly intensified microbial carbon limitation and shifted the community toward P limitation, forming a positive feedback loop. This study further indicated that the enhancement in the compositional constancy of the community driven by litter decomposition was essentially the result of synergistic interactions between resource competition and stress adaptation. Although the high enzymatic activities of BG and AKP during the initial decomposition stage aggravated diversity loss, the system ultimately maintained its robustness by selecting taxa that are highly tolerant to stress, such as Ascomycota, and functionally redundant microbial communities (Niu et al. 2025; Yan et al. 2025). While our controlled microcosm conditions cannot fully replicate the complexity of a natural salt marsh, they provide critical mechanistic insights into how litter chemistry drives these soil microbial processes. In the future, it will be necessary to quantify the relationships between decomposition rate, resource threshold, and compositional constancy response in field settings to optimize ecological regulation strategies for litter management in saline–alkali soils.

## 5 Conclusions

This controlled study clarifies the potential critical role of plant litter in the material cycling of salt marsh ecosystems, suggesting that species‐specific litter management could be a key consideration for ecological restoration, pending further field validation. Our findings reveal that leaf litter from halophytic plants drives soil ecological processes through differences in their chemical properties: decomposition significantly enhances soil nutrients, microbial activity, and community compositional constancy but exacerbates microbial carbon limitation and reduces the microbial nitrogen limitation. Key extracellular enzyme activities, microbial nutrient limitation status, and soil environmental factors are core mechanisms regulating this process.

## Author Contributions

**Peng Kang:** conceptualization (equal), methodology (equal), supervision (equal), writing – review and editing (equal). **Yaqing Pan:** conceptualization (equal), methodology (equal), resources (lead), supervision (equal), writing – original draft (lead), writing – review and editing (equal). **Bahetijiang Ayala:** data curation (equal), formal analysis (lead), investigation (equal). **Qing Wang:** data curation (equal), investigation (equal), software (lead). **Zixuan Chen:** data curation (equal), investigation (equal).

## Funding

This work was supported by the Xinjiang Natural Science Foundation Project (2025D01A56), National Natural Science Foundation of China (32360426), and the Doctoral Scientific Research Foundation of Xinjiang Normal University (XINUZBS2527).

## Conflicts of Interest

The authors declare no conflicts of interest.

## Supporting information


**Figure S1:** Sampling diagram.

**Figure S2:** Leaf quality in different halophytes.

**Figure S3:** The linear relationship between the AVD index of soil bacteria, and fungi and microbial nutrient limitation.

**Figure S4:** The explanatory rate of the dominant phyla under leaf decomposition of different halophyte for bacterial (A) and fungal AVD (B).

**Table S1:** Soil physicochemical properties under leaf decomposition of different halophyte.

## Data Availability

Sequence data are available through the Sequence Read Archive of Genbank under BioProject number PRJNA11413943 and PRJNA11413947. Soil physicochemical properties data are uploaded as tables.
