# Supplementary figures and images for: Halophyte Litter Decomposition Shapes Soil Microbial Community Compositional Constancy by Regulating Resource Stoichiometry and Enzymatic Activity in a Microcosm Study
Source: Ecol Evol. 2026 Jun 18;16(6):e73871. doi: 10.1002/ece3.73871 (PMC13279633; doi:10.1002/ece3.73871)

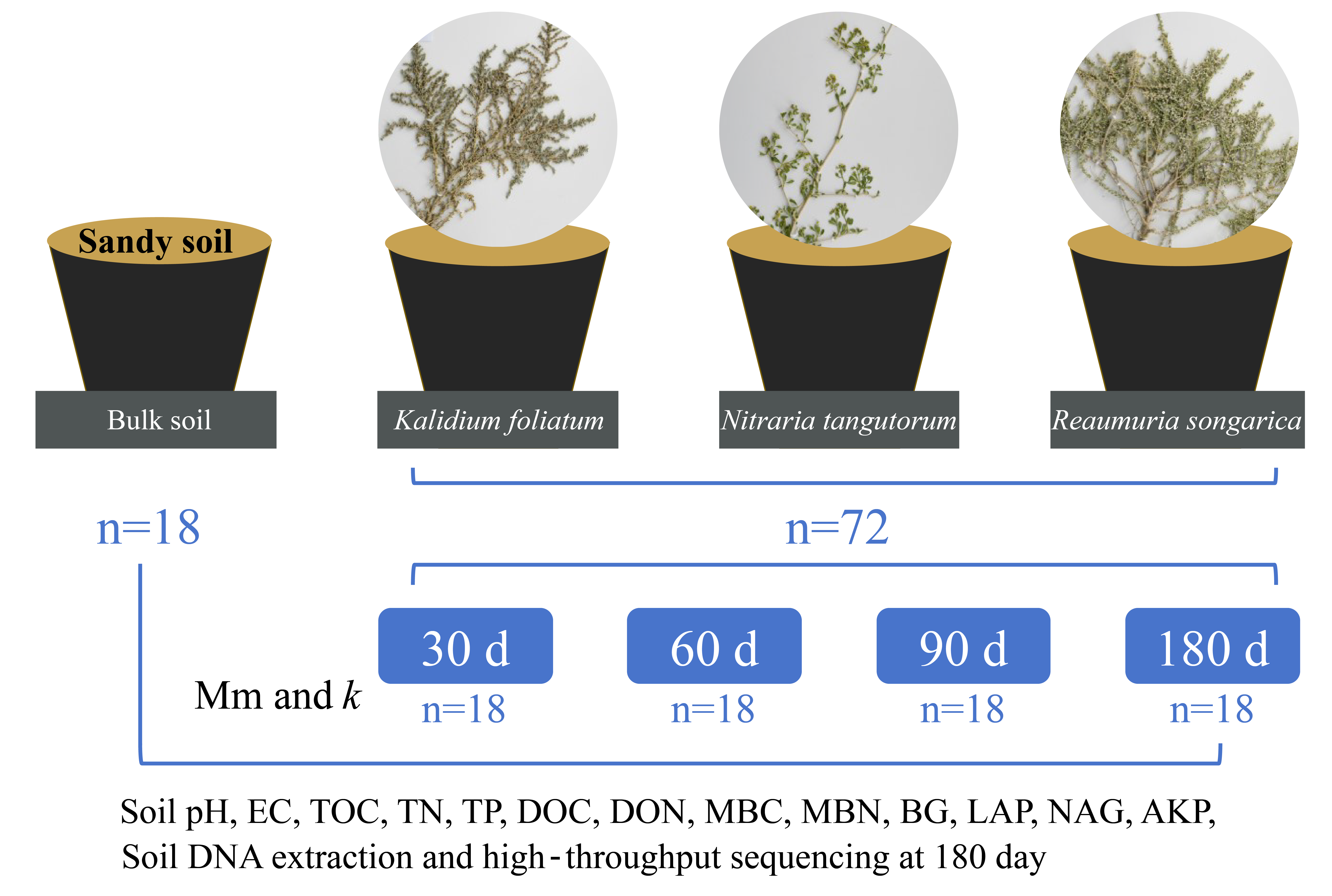

Supplement: Supplementary file 1 — Figure S1: Sampling diagram. [file ECE3-16-e73871-s004.tif]

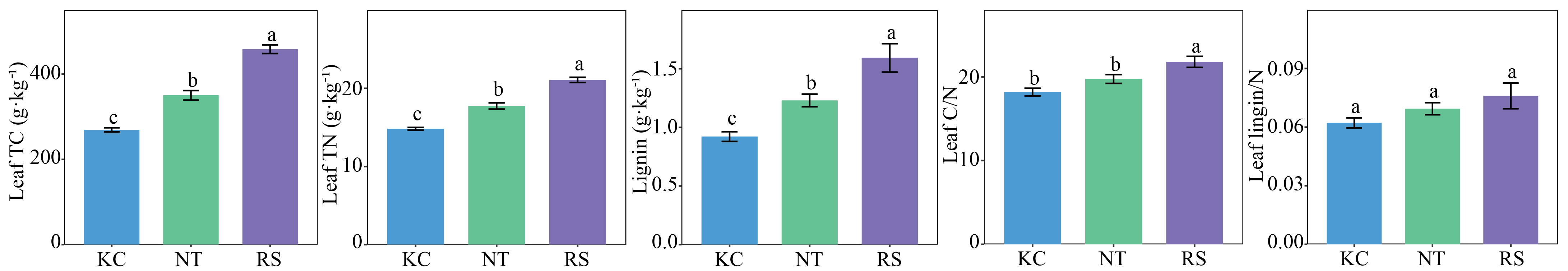

Supplement: Supplementary file 2 — Figure S2: Leaf quality in different halophytes. [file ECE3-16-e73871-s003.tif]

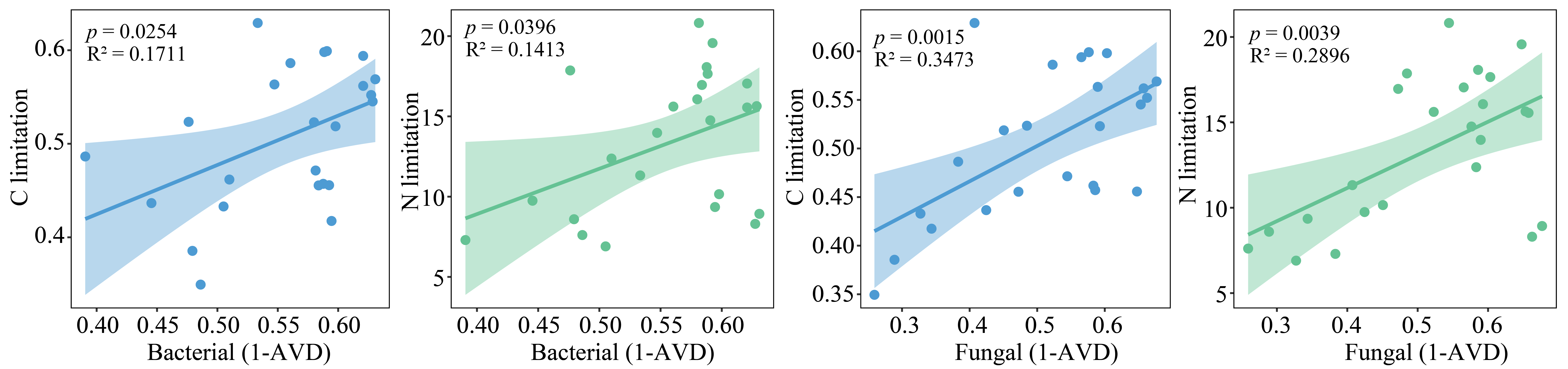

Supplement: Supplementary file 3 — Figure S3: The linear relationship between the AVD index of soil bacteria, and fungi and microbial nutrient limitation. [file ECE3-16-e73871-s001.tif]

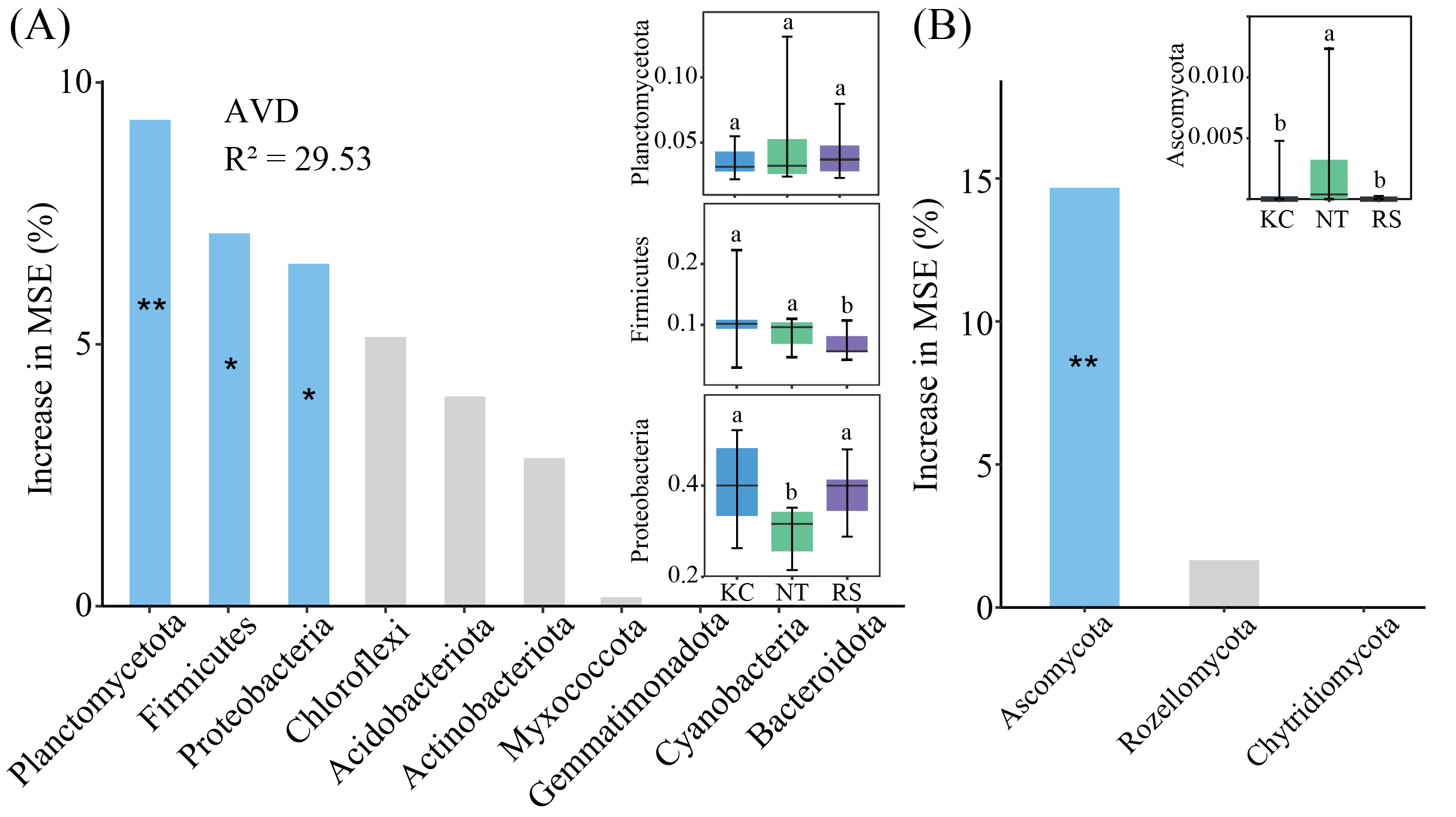

Supplement: Supplementary file 4 — Figure S4: The explanatory rate of the dominant phyla under leaf decomposition of different halophyte for bacterial (A) and fungal AVD (B). [file ECE3-16-e73871-s002.tif]
