# Supplementary material for: Halophyte Litter Decomposition Shapes Soil Microbial Community Compositional Constancy by Regulating Resource Stoichiometry and Enzymatic Activity in a Microcosm Study
Source: Ecol Evol. 2026 Jun 18;16(6):e73871. doi: 10.1002/ece3.73871 (PMC13279633; doi:10.1002/ece3.73871)
Supplement: Supplementary file 5 — Table S1: Soil physicochemical properties under leaf decomposition of different halophyte. [file ECE3-16-e73871-s005.pdf]

Table S1 Soil physicochemical properties under leaf decomposition of different halophyte.

Physicochemical properties	BS	KC	NT	RS
pH	8.80±0.07**b**	8.99±0.03**a**	8.89±0.04**ab**	8.75±0.05**b**
EC (μs·cm^{-1})	108.63±4.09**b**	145.40±7.37**a**	142.87±4.87**a**	119.83±3.85**b**
TOC (g·kg^{-1})	1.03±0.07**b**	1.63±0.10**a**	1.67±0.18**a**	2.06±0.20**a**
TN (g·kg^{-1})	0.09±0.01**c**	0.14±0.01**b**	0.16±0.01**b**	0.21±0.02**a**
TP (g·kg^{-1})	0.10±.01**b**	0.11±0.01**ab**	0.13±0.01**a**	0.12±0.01**a**
DOC (mg·kg^{-1})	14.76±0.37**c**	21.31±0.82**b**	25.22±0.52**b**	47.89±2.77**a**
DON (mg·kg^{-1})	1.12±0.07**c**	1.40±0.04**bc**	1.83±0.10**b**	3.81±0.34**a**
MBC (mg·kg^{-1})	33.66±1.02**d**	48.09±1.79b	54.88±1.89**b**	63.93±3.39**a**
MBN (mg·kg^{-1})	2.27±0.11**b**	3.25±0.25**a**	3.47±0.24**a**	3.84±0.18**a**
BG (nmol·g^{-1}·h^{-1})	5.93±0.01**d**	17.95±0.02**c**	18.98±0.03**b**	24.98±0.03**a**
LAP (nmol·g^{-1}·h^{-1})	92.63±7.18**b**	111.64±10.22**b**	123.04±13.95**ab**	172.25±31.34**a**
NAG (nmol·g^{-1}·h^{-1})	2.89±0.01**b**	2.93±0.01**a**	2.92±0.01**ab**	2.94±0.01**a**
AKP (nmol·g^{-1}·h^{-1})	8.56±0.68**b**	17.94±1.71**a**	20.41±2.08**a**	19.75±0.40**a**

Note: The data were presented as mean ± standard error (n = 6). Different letters in each column indicate significant differences at the $p < 0.05$ level (Duncan test).

For Review Only
